# Evaluating Chain-of-Thought reasoning in large language models for thyroid ultrasound interpretation: a dual-information approach

**DOI:** 10.3389/frai.2026.1780373

**Published:** 2026-03-23

**Authors:** Yu-Tong Zhang, Si-Yi Wu, Dong Zhang, Zheng-Yi Yang, Sheng-Wei Zhao, Hong-Cheng Han, Xin Yuan, Li-Rong Wang, Jiang Jue, Shao-Yi Du, Qi Zhou, Juan Wang

**Affiliations:** 1 The Department of Ultrasound, The Second Affiliated Hospital of Xi'an Jiaotong University, Xi'an, Shaanxi, China; 2 The Institute of Artificial Intelligence and Robotics, Xi'an Jiaotong University, Xi'an, Shaanxi, China

**Keywords:** ACR-TIRADS, Chain-of-Thought reasoning, dual-modal ultrasound characteristics, large language models, thyroid nodules

## Abstract

**Objective:**

To assess whether reasoning-capable large language models (LLMs) can accurately interpret both qualitative and quantitatively encoded ultrasound features of thyroid nodules within the ACR-TIRADS framework and improve diagnostic reliability.

**Methods:**

This retrospective study analyzed thyroid nodules with both radiologist-labeled qualitative ultrasound features and quantitatively encoded descriptors generated through standardized numerical modeling. Both formats were converted into structured prompts and input separately into four CoT-enabled LLMs (ChatGPT-O3, Grok-3, DeepSeek-R1, Gemini-2.5 Pro), each performing three reasoning rounds per task. Diagnostic performance was evaluated by accuracy and reproducibility, and two types of inconsistencies—cross-threshold and cross-modal conflicts—were quantified. Reasoning authenticity and conciseness were independently assessed by radiologists of varying experience. Sankey diagrams were used to summarize ACR-TIRADS category transitions.

**Results:**

ChatGPT-O3, Gemini-2.5 Pro, and Grok-3 showed strong ACR-TIRADS accuracy (91, 96, 96%), outperforming DeepSeek-R1 (79%). Grok-3 was highest in score-based accuracy (96%); DeepSeek-R1 lowest (52%). Reproducibility for categorization was Grok-3 93%, Gemini-2.5 Pro 90%, ChatGPT-O3 88%, vs. DeepSeek-R1 67%. For scoring reproducibility, Grok-3 (93%), ChatGPT-O3 (90%), and Gemini-2.5 Pro (79%) exceeded DeepSeek-R1 (18%). Physicians rated Grok-3 and Gemini-2.5 Pro highest in reasoning authenticity, while ChatGPT-O3 was most concise (mean 144 words). For quantitative tasks, Gemini-2.5 Pro (78%) and DeepSeek-R1 (74%) were most accurate; Grok-3 lowest (64%). Reproducibility was highest for Gemini-2.5 Pro (84%) and DeepSeek-R1 (86%). Across models, the proportion of nodules exhibiting cross-threshold discrepancies ranged from 3 to 17%, with Grok-3 lowest and DeepSeek-R1 highest. Cross-modal conflicts were more frequent, ranging from 27 to 36% across the four LLMs.

**Conclusion:**

Grok-3 excelled in qualitative tasks, while Gemini-2.5 Pro and DeepSeek-R1 showed strengths in quantitative analysis. CoT-enabled LLMs offered interpretable reasoning with promise for clinical decision support.

## Highlights

- Systematic comparison of four Chain-of-Thought LLMs with a unified diagnostic framework for thyroid ultrasound under ACR-TIRADS.
- Dual-modal evaluation using both qualitative ACR-TIRADS features and quantitatively encoded ultrasound descriptors.
- Multi-dimensional assessment covering accuracy, reproducibility, and the authenticity and conciseness of reasoning.
- Novel analysis of cross-threshold and cross-modal conflicts revealing key stability gaps in LLM-based diagnosis.

## Introduction

The rapid advancement of large language models (LLMs) has opened new frontiers in medical diagnostics, demonstrating unprecedented capabilities in data interpretation and clinical decision support (Reddy, 2024; Du et al., 2026; Onder et al., 2024). State-of-the-art LLMs, represented by ChatGPT, Claude, are capable of processing complex multimodal inputs and performing advanced reasoning, making them increasingly explored for specialized medical applications (Onder et al., 2024; Anthropic, 2024; OpenAI, 2024; Tordjman et al., 2025). In the field of radiology, where imaging interpretation plays a central role, these models have shown potential not only in standardizing report structures (Jiang et al., 2024; Subramaniam et al., 2025; Hu et al., 2025) but also in analyzing contextual relationships within medical texts, synthesizing information to support diagnostic decision-making with high accuracy (Raghunathan et al., 2025; Wang et al., 2024; Huang et al., 2024).

Thyroid nodule assessment is a particularly 6–7 important diagnostic area where accurate imaging interpretation is essential. Ultrasonography remains the cornerstone of initial evaluation and risk stratification, guided by the standardized American College of Radiology Thyroid Imaging Reporting and Data System (ACR-TIRADS) (Haugen et al., 2016), which classifies nodules based on sonographic features such as echogenicity, margins, and calcifications. Although previous studies have evaluated the ability of LLMs to interpret these qualitative features (Jiang et al., 2024; Alarifi, 2025; Kaba et al., 2024; Fung et al., 2025), earlier models were often limited by insufficient reasoning capacity and poor handling of structured imaging data. These limitations resulted in fragmented understanding, hallucinated conclusions, and suboptimal communication of findings.

The emergence of next-generation LLMs—such as ChatGPT-O3 and DeepSeek-R1—has led to notable improvements in logical reasoning, particularly through the adoption of Chain-of-Thought (CoT) prompting techniques (Tordjman et al., 2025; Wang et al., 2024; Maroncelli et al., 2024). These models can now more accurately interpret ACR-TIRADS descriptors and integrate structured clinical information with imaging features, enabling a more comprehensive diagnostic framework. Crucially, they begin to emulate the cognitive process of experienced clinicians, synthesizing sonographic characteristics, patient history, and numeric data to formulate clinically meaningful assessments. While qualitative features remain central to ultrasound interpretation, their subjectivity often introduces variability between observers.

This study provides the first systematic comparison of reasoning-capable, next-generation LLMs—ChatGPT-O3, Grok-3, DeepSeek-R1, and Gemini-2.5 Pro—in interpreting both descriptive and quantitatively encoded ACR-TIRADS ultrasound features derived from structured textual representations of ultrasound findings. By numerically standardizing selected ACR-TIRADS descriptors, we reduced subjectivity and created a unified testing framework that challenges LLMs with two fundamentally different text-based data modalities. Our findings reveal how these advanced models process qualitative versus quantitative ultrasound information at the feature-description level, highlighting their strengths, limitations, and potential roles in improving diagnostic accuracy and interpretability in thyroid nodule assessment.

## Methods

### Study design

This retrospective study was approved by the Institutional Review Board of our hospital (Approval No. 2022259; 2022), with a waiver of informed consent granted due to its retrospective design. A total of 100 patients (26 males and 74 females) diagnosed with a single thyroid nodule by ultrasonography were included, with a mean age of 46.68 ± 12.57 years. The benign or malignant nature of the nodules was determined either by histopathological examination (*n* = 73) or by clinical follow-up of at least 2 years (*n* = 27). For nodules classified based on follow-up, benignity was defined by imaging stability or regression without suspicious progression during the follow-up period.

**Figure 1:**
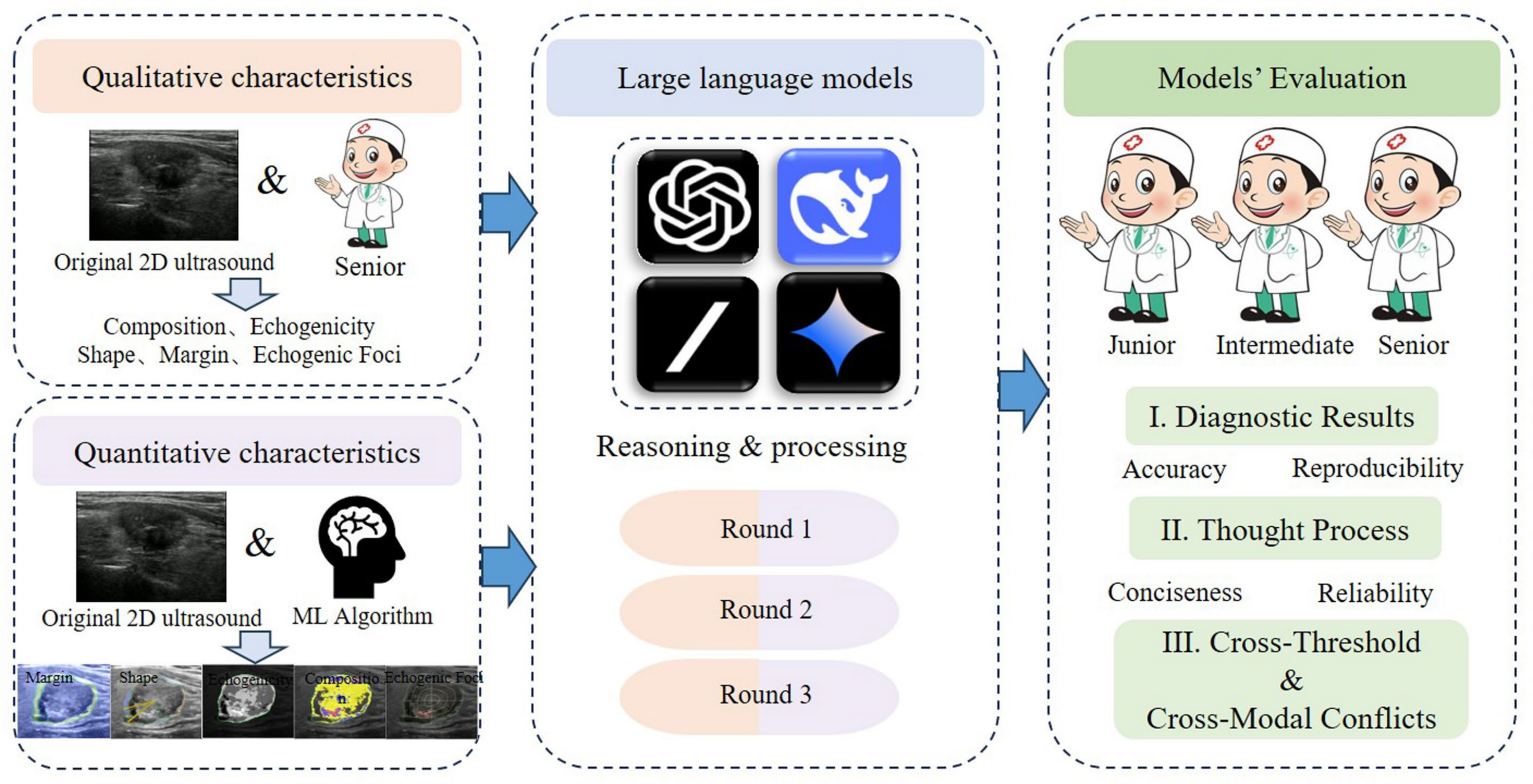
Overview of the study design for CoT-enabled LLMs.

To ensure diagnostic and educational representativeness, the cohort was selected to reflect a balanced distribution of benign and malignant nodules, with a wide spectrum of sonographic appearances and lesion sizes. Among these, 51 nodules were benign and 49 were malignant. The median maximum diameter of the nodules was 12 mm [interquartile range (IQR): 8–21.5 mm]. The dataset was reformatted to include detailed ultrasound reports, structured textual descriptions of sonographic features, and corresponding quantitative parameters extracted via machine learning (ML) algorithms. These characteristics made it a suitable dataset for benchmarking LLM performance in thyroid nodule diagnosis. Each LLM was treated as an independent “reader” and assessed across two complementary diagnostic pathways—qualitative evaluation (text-based ultrasound feature interpretation) and quantitative evaluation (ML-based numerical feature prediction)—allowing comparison of reasoning processes and diagnostic accuracy under standardized conditions. This study was conducted and reported in accordance with the TRIPOD-LLM guideline, and the corresponding checklist is provided in Supplementary material. The overall study workflow is illustrated in Figure 1.

### Qualitative ultrasound characteristics of thyroid nodules

Ultrasound examinations and independent evaluations of the nodules were performed by two experienced sonographers with 15 and 17 years of expertise in thyroid imaging, respectively. Each sonographer independently assessed a portion of the cases. In cases of uncertainty, the two sonographers discussed the findings; if a disagreement remained, a third senior sonographer with 26 years of thyroid ultrasound experience re-evaluated the nodule to reach a final decision. The grayscale ultrasonographic characteristics of thyroid nodules were assessed and documented in accordance with the guidelines of ACR-TIRADS. The baseline characteristics of the patients, including demographic and clinical ultrasound characteristics, are summarized in Table 1.

**Table 1:** Demographic and gray-scale ultrasound characteristics of benign and malignant thyroid nodules.

Characteristic	Overall (*n* = 100)	Benign (*n* = 51)	Malignant (*n* = 49)	*p*
Age (mean ± SD)	46.68 ± 12.57	50.10 ± 11.97	43.12 ± 12.31	0.005*
Sex, *N* (%)				
Male	26 (26)	13 (25.49)	13 (26.53)	0.91
Female	74 (74)	38 (74.51)	36 (73.47)	
Maximum diameter (mm)	12 (8, 21.50)	12 (8, 24)	11 (8, 19)	0.50
Location, *n* (%)				
Left lobe	46 (46)	22 (43.14)	24 (48.98)	0.73
Right lobe	50 (50)	26 (50.98)	24 (48.98)	
Isthmus	4 (4)	3 (5.88)	1 (2.04)	
Aspect ratio, *n* (%)				
<1	76 (76)	40 (78.43)	36 (73.47)	0.56
≥1	24 (24)	11 (21.57)	13 (26.53)	
Shape, *n* (%)				
Regular	48 (48)	27 (52.94)	21 (42.86)	0.31
Irregular	52 (52)	24 (47.06)	28 (57.14)	
Margin, *n* (%)				
Smooth	39 (39)	24 (47.06)	15 (30.61)	0.09
Ill-defined	61 (61)	27 (52.94)	34 (69.39)	
Composition, *n* (%)				
Cyst/A-Cystic/Spongiform	1 (1)	1 (1.96)	0 (0)	0.40
Cystic-solid mixed	9 (9)	6 (11.77)	3 (6.12)	
Solid or almost solid	90 (90)	44 (86.27)	46 (93.88)	
Echogenicity, *n* (%)				
Iso/hyperechoic	16 (16)	10 (19.61)	6 (12.25)	0.58
Hypoechoic	79 (79)	38 (74.51)	41 (83.67)	
Markedly hypoechoic	5 (5)	3 (5.88)	2 (4.08)	
Echogenic foci, *n* (%)				
None	49 (49)	34 (66.67)	15 (30.61)	0.003*
Macrocalcifications	8 (8)	2 (3.92)	6 (12.25)	
Rim calcifications	6 (6)	2 (3.92)	4 (8.16)	
Punctate echogenic foci	37 (37)	13 (25.49)	24 (48.98)	

*N*, number of patients; *n*, number of nodules; SD, standard deviation; *Statistically significant.

For subsequent LLM-based analysis, all qualitative ultrasound findings were converted into structured textual descriptions following a standardized template (e.g., “The following ultrasound features of a thyroid nodule: Nodule’s size: 7*8 mm, Taller-than-wide, Regular, Ill-defined, almost entirely solid, Hypoechoic, None Echogenic Foci.”). This ensured that the models received consistent, human-readable diagnostic narratives, enabling direct comparison between LLM interpretations and expert-derived ACR-TIRADS categorizations.

### Interpretation of quantitative ultrasound characteristics

Quantitative ultrasound characteristics were derived using a previously developed ML-based framework designed to mathematically encode grayscale sonographic features of thyroid nodules. This framework integrates automated nodule segmentation with uncertainty modeling, followed by quantitative reconstruction of sonographic descriptors corresponding to the ACR-TIRADS categories. Detailed methods are provided in Supplementary methods.

Briefly, multiple quantitative parameters were extracted to represent six core sonographic dimensions—shape, margin, composition, echogenicity, calcification, and aspect ratio. To address feature redundancy and high dimensionality, principal component analysis was applied for feature consolidation. The retained principal components were subsequently incorporated into logistic regression models to generate dimension-specific quantitative scores, yielding a single numerical value for each sonographic dimension per nodule.

To facilitate interpretability and integration into LLM-based reasoning, these machine-derived quantitative variables were further translated into semantically meaningful natural language descriptors (e.g., “strongly positively correlated,” “positively correlated,” “negatively correlated,” and “strongly negatively correlated”) based on their associations with pathological outcomes. This mapping enabled alignment between numerical imaging representations and text-based diagnostic reasoning within the LLM evaluation framework.

### Standardized evaluation protocol for model selection and interaction design

Given the rapid evolution of LLMs, model selection aimed to achieve balanced representation across major international and domestic developers while ensuring reproducibility and practical feasibility. Four representative general-purpose LLMs were evaluated: ChatGPT-O3 (OpenAI, San Francisco, CA, USA), Grok-3 (xAI, Palo Alto, CA, USA), DeepSeek-R1 (DeepSeek, Hangzhou, China), and Gemini-2.5 Pro (Google DeepMind, Mountain View, CA, USA).

Gemini-2.5 Pro (preview), ChatGPT-O3 (2025-04-16), and Grok-3 were accessed via their official public web interfaces, reflecting real-world usage by general clinical practitioners, whereas DeepSeek-R1 was accessed through API calls. All models were evaluated between February and May 2025 to minimize performance variation due to model updates. Gemini, ChatGPT, and Grok were operated under default out-of-the-box configurations, with sampling parameters and system prompts preset by the providers and not user-modifiable. For ChatGPT-O3, reasoning strength was set to “Auto,” while Grok-3 was operated in “Regular Mode.” For DeepSeek-R1, all sampling parameters (temperature 1.0, top-p 0.95) and the maximum generation length (64,000 tokens) were explicitly specified or followed official defaults to ensure parameter determinism. All models were queried without external plug-ins or real-time internet access, and responses reflected each model’s pre-trained knowledge at the time of evaluation.

A unified zero-shot prompting strategy with a predefined, stepwise interaction flow was applied, with each query initiated in a new chat session to avoid contextual carryover. Each prompt was repeated three times under identical conditions to assess output consistency, yielding a total of 2,400 responses across all models and experimental settings. The full prompts for both qualitative and quantitative evaluations are provided verbatim in Supplementary material.

### Evaluation criteria and performance assessment

The performance of the four LLMs was evaluated along two complementary dimensions: diagnostic outcome and reasoning process. For outputs derived from qualitative ultrasound characteristics, model-generated ACR-TIRADS scores and categories were compared with those assigned by experienced radiologists. For outputs derived from quantitative characteristics, diagnostic accuracy was determined using histopathological results as the reference standard. Reproducibility was operationally defined as output consistency across repeated runs and evaluated on a three-level scale: (1) high reproducibility, defined as identical outputs across all three repetitions; (2) moderate reproducibility, where two of three responses were consistent; and (3) low reproducibility, where one or none of the responses matched. This definition was used to enable comparative assessment of model output stability rather than to establish absolute reproducibility. For qualitative data, reproducibility was further analyzed separately for both ACR-TIRADS scores and categorical classifications.

Regarding the reasoning process, conciseness was quantified by calculating the word count of each model’s generated CoT, with shorter explanations reflecting greater succinctness. The authenticity of the reasoning—defined as the clinical plausibility and realism of the model’s interpretive logic—was independently assessed by three radiologists with 1, 5, and 10 years of experience, respectively, using a 5-point Likert scale (0 = entirely implausible; 5 = highly realistic). To ensure representative sampling, 10% of the total model outputs for each diagnostic modality were randomly selected using a fixed random seed, yielding 240 responses for reasoning evaluation.

A detailed summary of all evaluation indicators, scoring definitions, and methodological structure is presented in Supplementary Table 1.

### Statistical analysis

Patient demographic data and grayscale ultrasound characteristics were compared between benign and malignant groups using the independent-samples t-test, chi-square test, or Mann–Whitney U test, as appropriate. Continuous variables were expressed as mean ± SD or median (IQR), and categorical variables were presented as percentages. A *p*-value < 0.05 was considered statistically significant.

For model-based comparisons, the accuracy and reproducibility of ACR-TIRADS scores and categorizations among the four LLMs were analyzed using the Friedman test for paired data, followed by Wilcoxon signed-rank tests for *post hoc* pairwise comparisons. The reproducibility of model outputs for quantitative characteristics was similarly evaluated using the Friedman test. To capture model performance across repeated runs, we aggregated the results from three runs into an ordinal scale (reproducible accuracy score) ranging from 0 to 3. This approach effectively transformed repeated measures into a metric of model robustness and employed the Friedman test and Wilcoxon signed-rank test, which are suitable for paired ordinal data.

For the reasoning process, the authenticity and conciseness scores were compared among models using the Kruskal-Wallis H test, with *post hoc* pairwise analyses performed using Dunn’s test. All statistical analyses were performed using SPSS software (version 27.0.1.0; IBM Corp., Chicago, IL, USA).

## Results

### Accuracy of CoT-capable LLMs on qualitative and quantitative characteristics

For qualitative ACR-TIRADS scoring accuracy, the Friedman test revealed a significant overall difference among models (*p* < 0.001). Grok-3 achieved the highest accuracy (96%), followed by ChatGPT-O3 (92%) and Gemini-2.5 Pro (90%). Pairwise comparisons with Benjamini–Hochberg (BH) correction showed that Grok-3 performed significantly better than both Gemini-2.5 Pro (*p* = 0.005) and ChatGPT-O3 (*p* = 0.034). Gemini-2.5 Pro and ChatGPT-O3 formed a second performance tier, with no significant difference between them (*p* = 0.705). In contrast, all three models significantly outperformed DeepSeek-R1, which showed substantially lower accuracy (52%; all corrected *p* < 0.001).

Similarly, for ACR-TIRADS categorical classification, Grok-3 and Gemini-2.5 Pro achieved the highest accuracy (both 96%), followed by ChatGPT-O3 (91%) and DeepSeek-R1 (79%). Overall differences among models were statistically significant (*p* < 0.001). Pairwise comparisons with BH correction indicated that Gemini-2.5 Pro, Grok-3, and ChatGPT-O3 formed a high-performance tier, with no significant differences observed within this group (all corrected *p* > 0.05). In contrast, DeepSeek-R1 performed significantly worse than all other models (all corrected *p* < 0.001).

**Figure 2:**
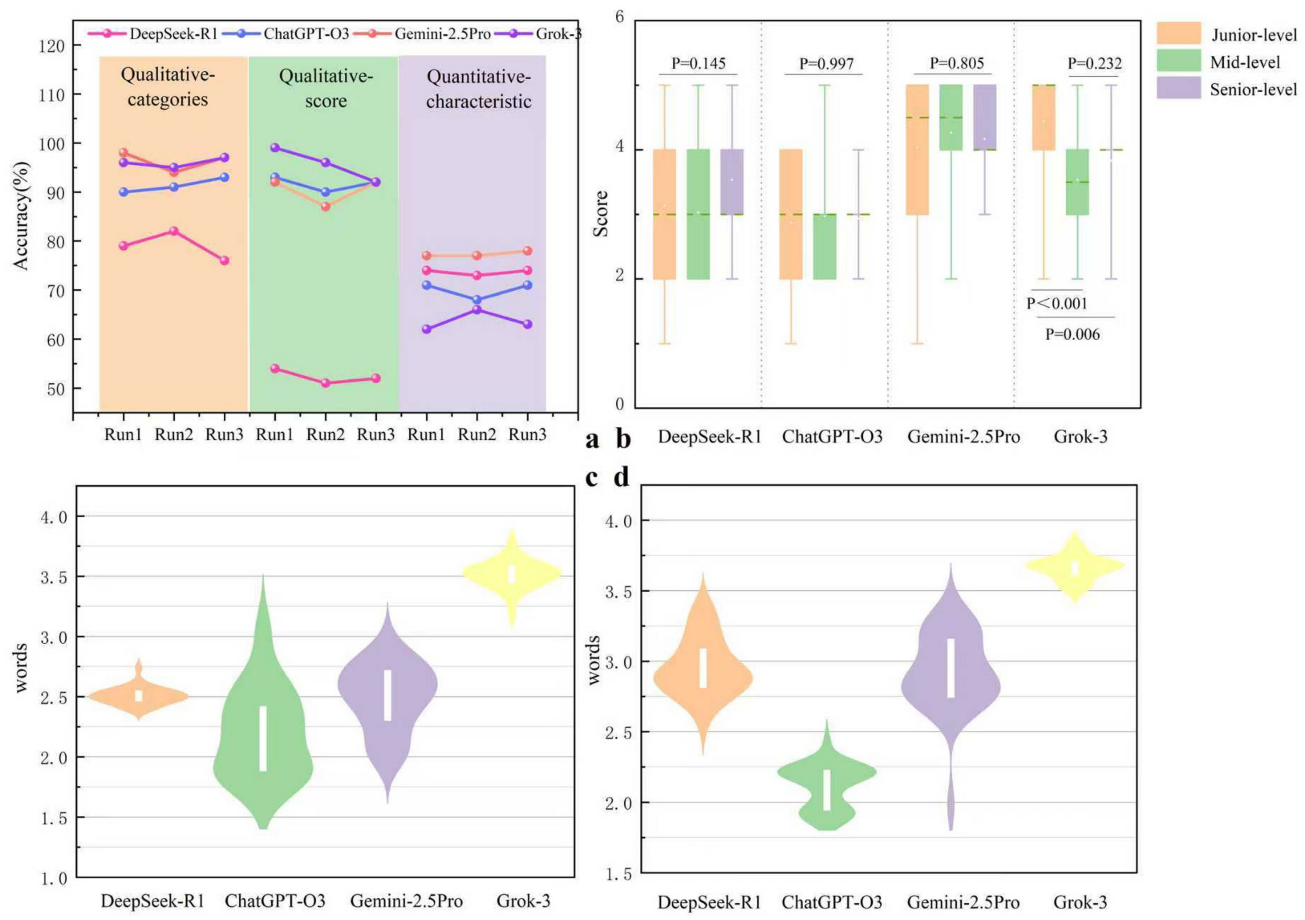
Comparative analysis of reasoning and performance in LLMs. **(a)** Describes the accuracy performance of each model across three separate runs. The accuracy is evaluated on both quantitative and qualitative characteristics, the latter of which comprises ACR-TIRADS categories and scores. **(b)** Compares the scores given to each model’s reasoning process by doctors of varying seniority levels (junior, mid, and senior). **(c,d)** Compare, respectively, the reasoning conciseness of different models for quantitative **(c)** and qualitative **(d)** words tasks (as measured by the log-transformed words count).

For quantitative feature–based prediction, overall model performance differed significantly (Friedman test, *p* < 0.001), with accuracy generally lower than that observed for qualitative interpretation. In terms of accuracy, Gemini-2.5 Pro achieved the highest performance (78%), followed by DeepSeek-R1 (74%), ChatGPT-O3 (70%), and Grok-3 (64%). Pairwise comparisons revealed that Gemini-2.5 Pro performed significantly better than Grok-3 (*p* < 0.001) and ChatGPT-O3 (*p* = 0.016). DeepSeek-R1 also demonstrated significantly higher accuracy than Grok-3 (*p* = 0.008). Although ChatGPT-O3 showed numerically higher accuracy than Grok-3, this difference did not reach statistical significance after BH correction (*p* = 0.053). No significant differences were observed between Gemini-2.5 Pro and DeepSeek-R1 or between DeepSeek-R1 and ChatGPT-O3 (both *p* > 0.05). Detailed accuracy results for both qualitative and quantitative tasks are summarized in Table 2 and Figure 2a.

**Table 2:** Comparison of qualitative and quantitative data across multiple performance metrics in four general-purpose LLMs.

			DeepSeek-R1			ChatGPT-O3		
Qualitative data, *n* (%)								
**Accuracy**	Run1	Run2	Run3	Total	Run1	Run2	Run3	Total
ACR-TIRADs categories	79/100 (79)	82/100 (82)	76/100 (76)	237/300 (79)	90/100 (90)	91/100 (91)	93/100 (93)	274/300 (91)
ACR-TIRADs score	54/100 (54)	51/100 (51)	52/100 (52)	157/300 (52)	93/100 (93)	90/100 (90)	92/100 (92)	275/300 (92)
**Reproducibility**	High	Moderate	Low		High	Moderate	Low	
ACR-TIRADs categories	67/100 (67)	30/100 (30)	3/100 (3)		88/100 (88)	12/100 (12)	0/100 (0)	
ACR-TIRADs score	18/100 (18)	64/100 (64)	18/100 (18)		90/100 (90)	10/100 (10)	0/100 (0)	
Quantitative data, *n* (%)								
**Accuracy**	Run1	Run2	Run3	Total	Run1	Run2	Run3	Total
	74/100 (74)	73/100 (73)	74/100 (74)	221/300 (74)	71/100 (71)	68/100 (68)	71/100 (71)	210/300 (70)
**Reproducibility**	High	Moderate	Low		High	Moderate	Low	
	86/100 (86)	14/100 (14)	0/100 (0)		77/100 (77)	23/100 (23)	0/100 (0)	
			Gemini-2.5 pro			Grok-3		
Qualitative data, *n* (%)								
**Accuracy**	Run1	Run2	Run3	Total	Run1	Run2	Run3	Total
ACR-TIRADs categories	98/100 (98)	94/100 (94)	97/100 (97)	289/300 (96)	96/100 (96)	95/100 (95)	97/100 (97)	288/300 (96)
ACR-TIRADs score	92/100 (92)	87/100 (87)	92/100 (92)	271/300 (90)	99/100 (99)	96/100 (96)	92/100 (92)	287/300 (96)
**Reproducibility**	High	Moderate	Low		High	Moderate	Low	
ACR-TIRADs categories	90/100 (90)	10/100 (10)	0/100 (0)		93/100 (93)	7/100 (7)	0/100 (0)	
ACR-TIRADs score	79/100 (79)	21/100 (21)	0/100 (0)		93/100 (93)	7/100 (7)	0/100 (0)	
Quantitative data, *n* (%)								
**Accuracy**	Run1	Run2	Run3	Total	Run1	Run2	Run3	Total
	77/100 (77)	77/100 (77)	78/100 (78)	232/300 (78)	62/100 (62)	65/100 (65)	63/100 (63)	190/300 (63)
**Reproducibility**	High	Moderate	Low		High	Moderate	Low	
	84/100 (84)	16/100 (16)	0/100 (0)		73/100 (73)	27/100 (27)	0/100 (0)	

*n*, number of nodules.

### Reproducibility of CoT-capable LLMs on qualitative and quantitative characteristics

Significant differences in reproducibility were observed among the four models for qualitative ACR TI-RADS categorization across three repeated runs (*p* < 0.001). Grok-3 demonstrated the highest stability, with 93% of outputs showing high reproducibility and 7% moderate reproducibility. In contrast, DeepSeek-R1 exhibited the lowest stability (67% high, 30% moderate, and 3% low reproducibility). ChatGPT-O3 (88% high, 12% moderate) and Gemini-2.5 Pro (90% high, 10% moderate) showed intermediate performance. Pairwise comparisons with BH correction indicated that Grok-3, Gemini-2.5 Pro, and ChatGPT-O3 formed a high-consistency tier, with no significant differences among them (all corrected *p* > 0.05). By contrast, DeepSeek-R1 demonstrated significantly lower reproducibility than all other evaluated models (all corrected *p* < 0.001).

For qualitative ACR-TIRADS scoring reproducibility, a similar pattern was observed, with overall differences again reaching statistical significance (*p* < 0.001). ChatGPT-O3 (90% high, 10% moderate), Gemini-2.5 Pro (79% high, 21% moderate), and Grok-3 (93% high, 7% moderate) formed a high-reproducibility group, whereas DeepSeek-R1 demonstrated markedly poorer stability (18% high, 64% moderate, and 18% low reproducibility). Pairwise comparisons with BH correction showed that Grok-3 and ChatGPT-O3 exhibited comparable reproducibility (*p* = 0.439), while Grok-3 demonstrated significantly higher reproducibility than Gemini-2.5 Pro (corrected *p* = 0.012). Reproducibility between Gemini-2.5 Pro and ChatGPT-O3 was numerically similar but did not reach statistical significance after correction (*p* = 0.058). Importantly, DeepSeek-R1 consistently exhibited significantly lower reproducibility than all other models (all corrected *p* < 0.001).

For quantitative predictions, reproducibility also differed among models (*p* = 0.041). DeepSeek-R1 achieved the highest reproducibility (86% high, 14% moderate), followed by Gemini-2.5 Pro (84% high, 16% moderate) and ChatGPT-O3 (77% high, 23% moderate). Grok-3 showed the lowest values (73% high, 27% moderate). However, after BH correction, no individual model differences remained statistically significant, suggesting that the overall effect reflected the cumulative influence of several modest differences rather than a single dominant contrast. A detailed breakdown of reproducibility metrics for both qualitative and quantitative tasks is presented in Table 2.

### Authenticity of CoT responses by reasoning-capable LLMs

For reasoning authenticity, the Kruskal-Wallis H test demonstrated highly significant differences in physician ratings among the four models (*p* < 0.001). Pairwise post-hoc Dunn tests with BH correction identified two distinct performance tiers. Grok-3 and Gemini-2.5 Pro received higher authenticity scores, with no significant difference between them (*p* = 0.284). ChatGPT-O3 and DeepSeek-R1 formed the lower-performing group, also without significant differences within the group (*p* = 0.26). However, all models in the higher-performing tier scored significantly higher than those in the lower tier (all corrected *p* < 0.001).

Notably, rater experience influenced authenticity ratings only for Grok-3: junior physicians assigned significantly higher scores to Grok-3 compared with mid-level (corrected *p* < 0.001) and senior radiologists (corrected *p* = 0.006). No such trend was observed for the other models. Detailed results are presented in Table 3 and Figure 2b.

**Table 3:** Comparison of multiple performance metrics in reasoning processes of four general-purpose LLMs by physicians with different levels of experience.

	Qualitative characteristics					Quantitative characteristics
	Authenticity				Conciseness	Conciseness
	Junior-level	Mid-level	Senior-level	*p*		
Deepseek-R1	3.0 (2.0, 4.3)	3.0 (2.0, 4.3)	3.0 (3.0, 4.0)	0.13	314.5 (285.3, 352.3)	824.5 (643.0, 1,236.8)
ChatGPT-O3	3.0 (2.0, 4.0)	3.0 (2.0, 3.3)	3.0 (2.0, 3.0)	1.00	144.0 (74.5, 282.0)	157.0 (87.3, 168.0)
Gemini-2.5 pro	4.5 (3.0, 5.0)	4.5 (4.0, 5.0)	4.0 (4.0, 5.0)	0.78	336.0 (193.8, 526.5)	766.5 (549.5, 1,481.8)
Grok-3	5.0 (4.0, 5.0)	3.5 (3.0, 4.0)	4.0 (3.8, 4.0)	<0.001^*^	3,376.0 (2,729.0, 3,852.8)	4,751.0 (4,074.3, 5,144.3)

* Statistically significant.

Inter-rater reliability among radiologists was further assessed using a two-way mixed-effects model with average-measure intraclass correlation coefficients (ICC). Overall, the aggregated ICC across all models was 0.634 (95% CI: 0.330–0.810; *p* < 0.001), indicating good reliability of the pooled ratings. Inter-rater agreement varied by model, with DeepSeek-R1 demonstrating excellent consensus (ICC = 0.787) and Grok-3 showing moderate agreement. In contrast, ChatGPT-O3 exhibited lower inter-rater reliability, suggesting that its reasoning outputs elicited more divergent interpretations among experts with different levels of experience. These findings support the validity of using aggregated scores for analysis while also highlighting subtle but meaningful differences in how experts perceive the reasoning characteristics of different large language models.

### Conciseness of CoT responses by reasoning-capable LLMs

For reasoning conciseness, significant differences were observed across models for both qualitative and quantitative tasks (both *p* < 0.001). For qualitative reasoning, ChatGPT-O3 produced the most concise explanations (median 144.0 words, IQR 74.5–282.0), followed by DeepSeek-R1 (median 314.5, IQR 285.3–352.3), Gemini-2.5 Pro (median 336.0, IQR 193.8–526.5), and Grok-3 (median 337.6, IQR 272.9–385.28; *p* < 0.001). Pairwise comparisons with BH correction showed that ChatGPT-O3 generated significantly shorter outputs than Gemini-2.5 Pro (*p* = 0.0096), DeepSeek-R1 (*p* = 0.0127), and Grok-3 (*p* < 0.001). DeepSeek-R1 and Gemini-2.5 Pro did not differ significantly in output length (*p* = 0.863), and both produced significantly shorter explanations than Grok-3 (both corrected *p* < 0.001).

For quantitative reasoning, ChatGPT-O3 again produced the shortest outputs (median 157.0, IQR 87.3–168.0), significantly more concise than all other models (all corrected *p* < 0.001). Gemini-2.5 Pro (median 766.5, IQR 549.5–1,481.8) and DeepSeek-R1 (median 824.5, IQR 643.0–1,236.8) demonstrated intermediate verbosity with no significant difference between them (*p* = 0.727), whereas Grok-3 generated the longest responses (median 4,751.0, IQR 4,074.3–5,144.3; *p* < 0.001 vs. all models). Comprehensive conciseness results for qualitative and quantitative reasoning are summarized in Table 3 and Figures 2c,d.

For quantitative prediction, all models demonstrated high sensitivity (0.92–0.98) but relatively low specificity (0.28–0.60). This performance profile indicates a strong ability to detect malignant nodules with a low risk of false negatives, albeit at the expense of increased false-positive classifications of benign nodules. Detailed performance metrics of the quantitative models are provided in Supplementary Table 2.

### Cross-threshold and cross-modal conflicts in LLM-based thyroid nodule diagnosis

Two categories of diagnostic inconsistencies were examined: cross-threshold conflicts in qualitative assessments and cross-modal conflicts between qualitative and quantitative diagnostic outputs.

**Figure 3:**
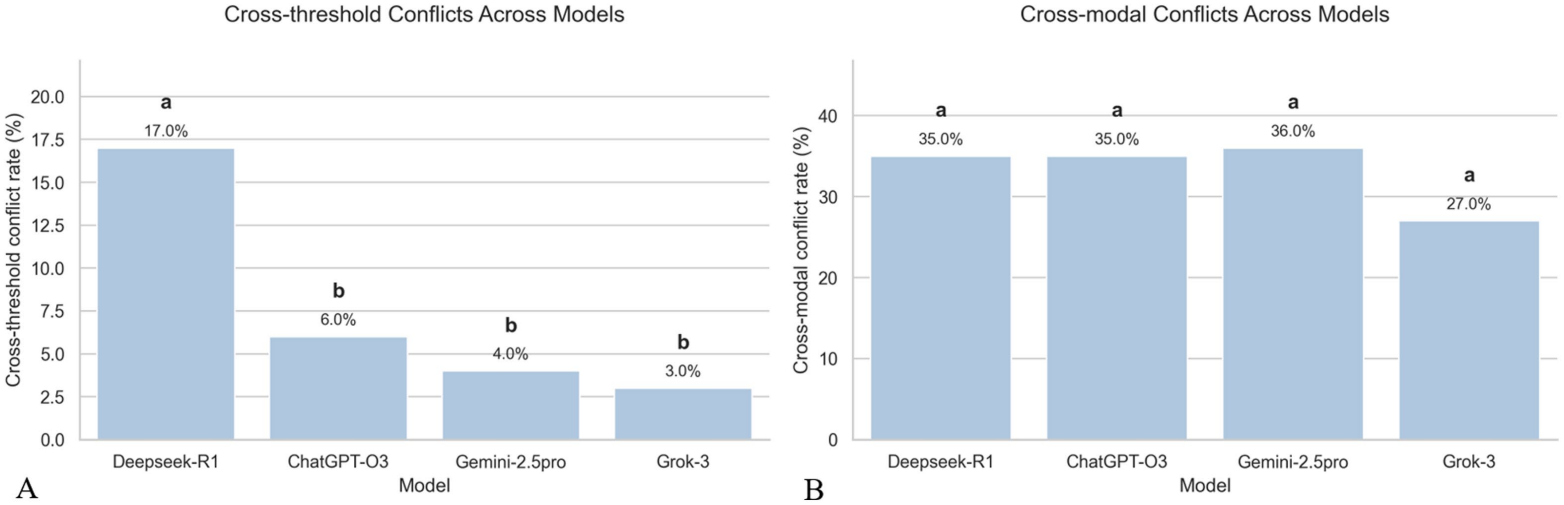
Conflict rates of four large language models. **(A)** Cross-threshold conflicts between qualitative and quantitative threshold-based outputs. **(B)** Cross-modal conflicts between qualitative and quantitative modalities. Bars show conflict rates with significance grouping letters derived from pairwise two-proportion *z*-tests. Models labeled with the same letter do not differ significantly (*p* ≥ 0.05); different letters indicate significant differences.

Cross-threshold conflicts were defined as instances where the ACR-TIRADS category assigned by an LLM crossed a clinically meaningful decision boundary relative to the expert rating (e.g., TR3 ↔ TR4 or TR4 ↔ TR5). Such shifts are clinically relevant because they may alter patient management decisions, such as whether to perform fine-needle aspiration. As shown in Figure 3A, the proportion of nodules exhibiting cross-threshold discrepancies ranged from 3 to 17% across the four LLMs (DeepSeek-R1 17%, ChatGPT-O3 6%, Grok-3 3%, Gemini-2.5 pro 4%). DeepSeek-R1 showed a significantly higher conflict rate than all other models (*p* < 0.05), whereas ChatGPT-O3, Gemini-2.5 Pro, and Grok-3 did not differ significantly from each other. These disagreements were mainly concentrated near the TR3–TR4 boundary, suggesting model sensitivity to borderline imaging features.

**Figure 4:**
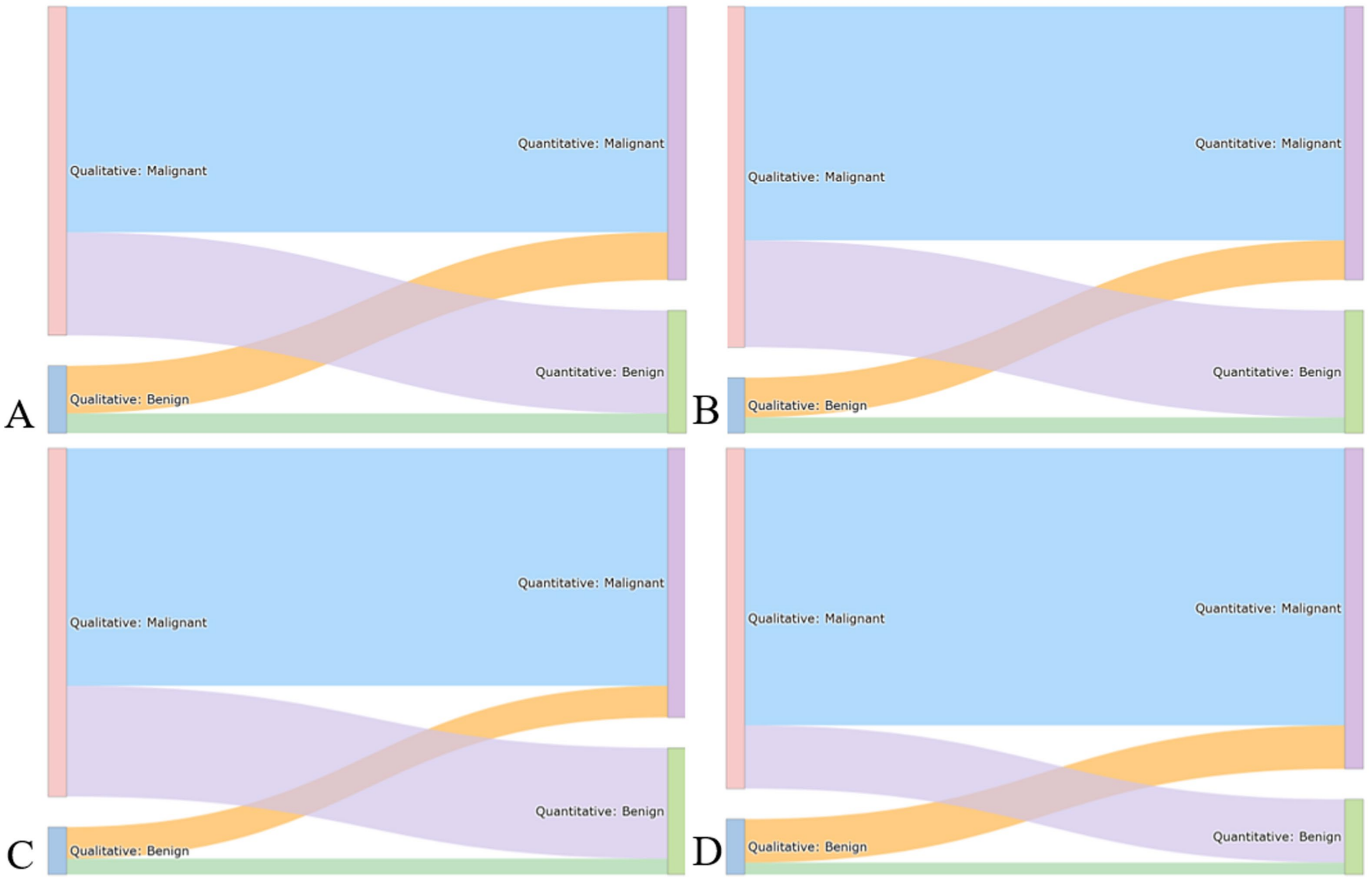
Sankey diagrams illustrate the flow between qualitative assessments and quantitative classifications for four large language models **(A–D)**. DeepSeek-R1 **(A)**, ChatGPT-O3 **(B)**, Gemini-2.5 Pro **(C)**, and Grok-3 **(D)**.

Cross-modal conflicts were defined as discordances between an LLM’s qualitative reasoning (text-based feature interpretation) and quantitative prediction (machine learning-based numerical estimation). A conflict was recorded when the qualitative interpretation implied benignity while the quantitative prediction indicated malignancy, or vice versa. The frequency of such conflicts ranged from 27 to 36% across models (DeepSeek-R1 35%, ChatGPT-O3 35%, Grok-3 27%, Gemini-2.5 pro 36%), as shown in Figure 3B. No statistically significant differences were observed among the four models (all *p* > 0.05). The overall distribution of these qualitative-quantitative discrepancies is visualized in a Sankey diagram (Figure 4), which illustrates the directional flow between the two diagnostic modalities and highlights the relative magnitude of each disagreement category.

## Discussion

This study systematically evaluated four CoT–enabled LLMs within the ACR-TIRADS framework using both qualitative and quantitatively encoded ultrasound features. To our knowledge, this is one of the few studies comparing model performance across these two fundamentally different data formats. The findings demonstrate that all four LLMs exhibit a certain level of feasibility in interpreting qualitative ultrasound features, with Grok-3 achieving the highest accuracy in both scoring and categorization, followed by ChatGPT-O3 and Gemini-2.5 Pro. In contrast, DeepSeek-R1 showed markedly inferior accuracy and reproducibility on qualitative tasks. However, for quantitative numerical reasoning, Gemini-2.5 Pro and DeepSeek-R1 outperformed the other models, suggesting that different feature types may evoke different reasoning mechanisms within LLMs. These results highlight the strong influence of data representation on model performance and suggest that future LLM applications may require task-specific input strategies, in line with recent evaluations of LLMs on structured, standardized radiological reasoning tasks (Camur et al., 2024).

The superior performance of Grok-3 on qualitative tasks may be attributed to its strong language-based reasoning architecture. Trained with extensive computational resources, Grok-3 exhibits advanced logical reasoning capabilities (Leucuța et al., 2025; Agarwal et al., 2025), enabling it to process descriptive ultrasound features—such as margins, shape, and echogenicity—in a manner more consistent with radiologists’ reasoning patterns. Nonetheless, its reasoning chains were often lengthy (median length: 3,376 words) and exhibited occasional hesitation when evaluating low-risk nodules (TIRADS category 2), which may impede clinical usability. Despite these limitations, its stable interpretation of ACR-TIRADS descriptors and fine-grained feature decomposition indicate that structured qualitative information remains the task type where current LLMs perform most reliably.

For quantitative features, mathematically modeled ACR-TIRADS descriptors were incorporated to reduce subjectivity and improve consistency. Unlike the qualitative tasks, DeepSeek-R1 and Gemini-2.5 Pro showed superior accuracy in numerical reasoning, suggesting that quantitative prediction relies more heavily on a model’s mathematical and symbolic computation capabilities rather than long-form linguistic reasoning. Grok-3’s reduced performance in this domain may indicate that its reasoning architecture is optimized for semantic logic rather than abstract numerical mapping. This divergence underscores the need for future medical LLMs to incorporate specialized modules tuned for different input modalities to ensure cross-task consistency.

Two clinically relevant diagnostic conflicts were also noted. DeepSeek-R1 showed the highest rate of cross-threshold inconsistencies (17%), whereas Grok-3 showed the lowest (3%), likely reflecting differences in how models weight subtle features such as mildly irregular margins or hypoechogenicity. Cross-modal conflicts were also frequent (27–36%). Beyond the inherent gap between semantic reasoning in qualitative tasks and mathematical processing in quantitative tasks, a key contributing factor may be that general-purpose LLMs have limited exposure to structured numerical ultrasound descriptors during pretraining. Because our quantitative features represent a novel encoding strategy, current models may not yet reason over them reliably. Future task-specific or fine-tuned models trained directly on such data are expected to improve consistency and overall diagnostic performance.

Previous studies have shown that LLMs can support structured reporting (Jiang et al., 2024; Wang et al., 2024), guideline adherence assessment (Alarifi, 2025; Xia et al., 2025), and clinical decision-making (Wu et al., 2024). However, most prior work has focused on conventional LLMs and has not deeply examined reasoning consistency or cross-modal diagnostic behavior. Our findings extend this literature by showing that CoT-enabled models provide more transparent reasoning chains, yet important limitations remain. ChatGPT-O3 frequently omitted intermediate logical steps, leading to gaps between reported features and final conclusions. Gemini-2.5 Pro often misestimated malignancy risk in TR4-level nodules and generated incomplete reasoning chains. DeepSeek-R1 demonstrated the lowest overall accuracy, with frequent errors in both feature scoring and malignancy probability estimation. Although all CoT models exhibited certain clinician-like reasoning patterns, their feature prioritization, risk weighting, and handling of ambiguous descriptors still diverged from radiologists’ cognitive processes. Even so, the ability of these models to engage in structured, interpretable reasoning represents a meaningful step toward more reliable and clinically aligned AI-assisted ultrasound diagnosis.

This study has several limitations. First, this was a single-center study with a relatively limited sample size, which may restrict statistical power for subgroup analyses and limit generalizability. The balanced benign–malignant distribution, while useful for model comparison, does not reflect real-world disease prevalence and may influence performance estimates. Second, only four representative Chain-of-Thought–enabled large language models were evaluated, which may not capture the full diversity of available LLMs. Third, the study relied on structured text inputs rather than raw ultrasound images; therefore, the findings apply specifically to text-based reasoning over encoded ultrasound features and may not directly translate to multimodal image-based applications. Future studies should validate these findings in larger, multi-center cohorts and explore multimodal LLMs in clinically realistic workflows.

## Conclusion

In summary, reasoning-capable LLMs show promising potential for assisting thyroid nodule assessment. Grok-3 performed best on qualitative features, while Gemini-2.5 Pro and DeepSeek-R1 showed advantages in quantitative analysis. Further validation using larger cohorts and image-based multimodal inputs is needed to enhance clinical reliability.

## Data Availability

The original contributions presented in the study are included in the article/Supplementary material, further inquiries can be directed to the corresponding authors.
