# Supplementary material for: Evaluating Chain-of-Thought reasoning in large language models for thyroid ultrasound interpretation: a dual-information approach
Source: Front Artif Intell. 2026 Mar 23;9:1780373. doi: 10.3389/frai.2026.1780373 (PMC13050882; doi:10.3389/frai.2026.1780373)
Supplement: Supplementary file 1 [file Data_Sheet_1.docx]

**Methods**

**Machine Learning for Quantitative Analysis of Thyroid Grayscale Ultrasound Images**

In this study, we built upon a previously developed machine learning framework for quantitative analysis of grayscale thyroid ultrasound images. The overall pipeline consisted of two major stages: automated nodule segmentation with uncertainty modeling, followed by quantitative feature reconstruction corresponding to ACR-TIRADS descriptors.

In the first stage, a hybrid self-supervised learning framework was employed to generate initial segmentation masks for thyroid nodules. This model was subsequently refined using a supervised fine-tuning strategy incorporating Monte Carlo–based sampling to model uncertainty in nodule boundary delineation. This approach enabled probabilistic characterization of segmentation confidence and provided a basis for subsequent quantitative feature extraction.

In the second stage, quantitative reconstruction of sonographic features was performed.

- **Aspect Ratio** was calculated based on the ratio of the long and short axes from a fitted elliptical contour of the nodule.
- **Clarity of Margin** was assessed by measuring the uncertainty error in the delineation of the nodule boundary.
- **Regularity of Shape** was quantified based on the degree of convexity and concavity in the nodule contour.
- **Echogenicity, Composition** was performed by analyzing the pixel intensity distributions and calculating the relative proportions within corresponding regions.
- **Calcification** was identified based on grayscale intensity differences using an adaptive thresholding strategy, with threshold selection guided by consistency with expert radiologist annotations.

Detailed algorithmic implementations and parameter settings are provided in a separate methodological study currently under submission.

**Design of Input Prompts for LLM Evaluation**

To ensure reproducibility and transparency, the exact prompts used for model evaluation are provided below. Two representative examples are included:

- **Qualitative Prompt (based on descriptive grayscale ultrasound features):**
  -Do you know ACR-TIRADs? The malignant risk of nodules can be classified as TR1, TR2, TR3, TR4, TR5?

-If you are a radiologist. Now a xx-year-old male/female patient comes for a consultation, with the following ultrasound features of a thyroid nodule: xxxxx. I hope you can provide a diagnosis for this nodule and perform an ACR-TIRADS classification.

- **Quantitative Prompt (based on numerically encoded ACR-TIRADS descriptors):**
  -I have a dataset in which 'GT' is a binary qualitative variable representing the benignity or malignancy of lesions ('0' for benign, '1' for malignant). 'Shape', 'Calcification', 'Composition', 'Echo', 'Margin', and 'Aspect_ratio' are six quantitative features extracted using mathematical methods, representing ultrasound characteristics of 'shape', 'calcification', 'composition', 'echogenicity', 'margin', and 'aspect ratio', respectively.

- 'Shape' is significantly positively correlated with 'GT': the larger the shape value, the more likely GT is 1.

- 'Calcification' is negatively correlated with GT but not statistically significant: although the coefficient is negative, it's uncertain whether it truly affects GT.

- 'Composition' is positively correlated with GT but not statistically significant: it remains unclear whether a larger composition value would lead to a higher likelihood of GT being 1.

- 'Echo' is significantly positively correlated with GT: the larger the echo value, the more likely GT is 1.

- 'Margin' is negatively correlated with GT but not statistically significant: it's uncertain whether a higher margin value affects GT.

- 'Aspect_ratio' is strongly negatively correlated with GT and is close to the threshold of statistical significance: the larger the aspect_ratio, the more likely GT is 0, but this relationship still requires further validation.

Now I will give you a set of data. Please judge the 'GT' value of each sample based on the above rules. Let me know when you're ready.

STable 1. Structured Evaluation Framework for Qualitative (Text-based) and Quantitative (Machine Learning–based) LLM Diagnosis of Thyroid Nodules

| Category | Evaluation Dimension | Indicator | Definition | Evaluation Method | Scoring |
| --- | --- | --- | --- | --- | --- |
| Qualitative Evaluation (Ultrasound Feature Text-based) | Diagnostic Accuracy | ACR-TIRADS Score | Compare model-generated TIRADS scores with expert radiologists |  |  |
|  |  | ACR-TIRADS Category | Compare model-generated TIRADS Categories with expert radiologists |  |  |
|  | Reproducibility | Score Stability | Evaluate consistency across 3 independent runs | 3-tier reproducibility: High (3/3 same), Moderate (2/3 same), Low (≤1 same) | High / Moderate / Low |
|  |  | Category Stability |  |  |  |
|  | Reasoning Process | Conciseness | Brevity of model-generated reasoning text | Word count of chain-of-thought | Fewer words = higher conciseness |
|  |  | Authenticity | Clinical realism of reasoning about ultrasound features | Rated by 3 radiologists (1, 5, 10 years’ experience) | 5-point Likert: 0–5 |
| Quantitative Evaluation (Machine Learning-based Quantitative Ultrasound Features) | Diagnostic Accuracy | Malignancy Probability Alignment | Binary (benign vs. malignant) consistency with pathology | ROC-AUC/  Accuracy/Sensitivity/Specificity |  |
|  | Reproducibility | Decision Consistency | Consistency of final benign/malignant classification | 3-tier reproducibility: High (3/3 same), Moderate (2/3 same), Low (≤1 same) | High / Moderate / Low |

STable 2. Quantitative Diagnostic Performance of Large Language Models

| Model | Accuracy | Sensitivity (Recall) | Specificity (Spec) | PPV (Precision) | NPV | F1-Score |
| --- | --- | --- | --- | --- | --- | --- |
| Deepseek-R1 | 0.750 (0.657-0.825) | 0.940 (0.838-0.979) | 0.560 (0.423-0.688) | 0.681 (0.564-0.779) | 0.903 (0.751-0.967) | 0.790 (0.708-0.864) |
| Grok-3 | 0.670 (0.573-0.754) | 0.980 (0.895-0.996) | 0.360 (0.241-0.499) | 0.605 (0.496-0.704) | 0.947 (0.754-0.991) | 0.748 (0.661-0.829) |
| Gemini-2.5pro | 0.780 (0.689-0.850) | 0.960 (0.865-0.989) | 0.600 (0.462-0.724) | 0.706 (0.589-0.801) | 0.938 (0.799-0.983) | 0.814 (0.736-0.882) |
| Chatgpt-o3 | 0.730 (0.636-0.807) | 0.920 (0.812-0.968) | 0.540 (0.404-0.670) | 0.667 (0.549-0.766) | 0.871 (0.711-0.949) | 0.773 (0.685-0.848) |


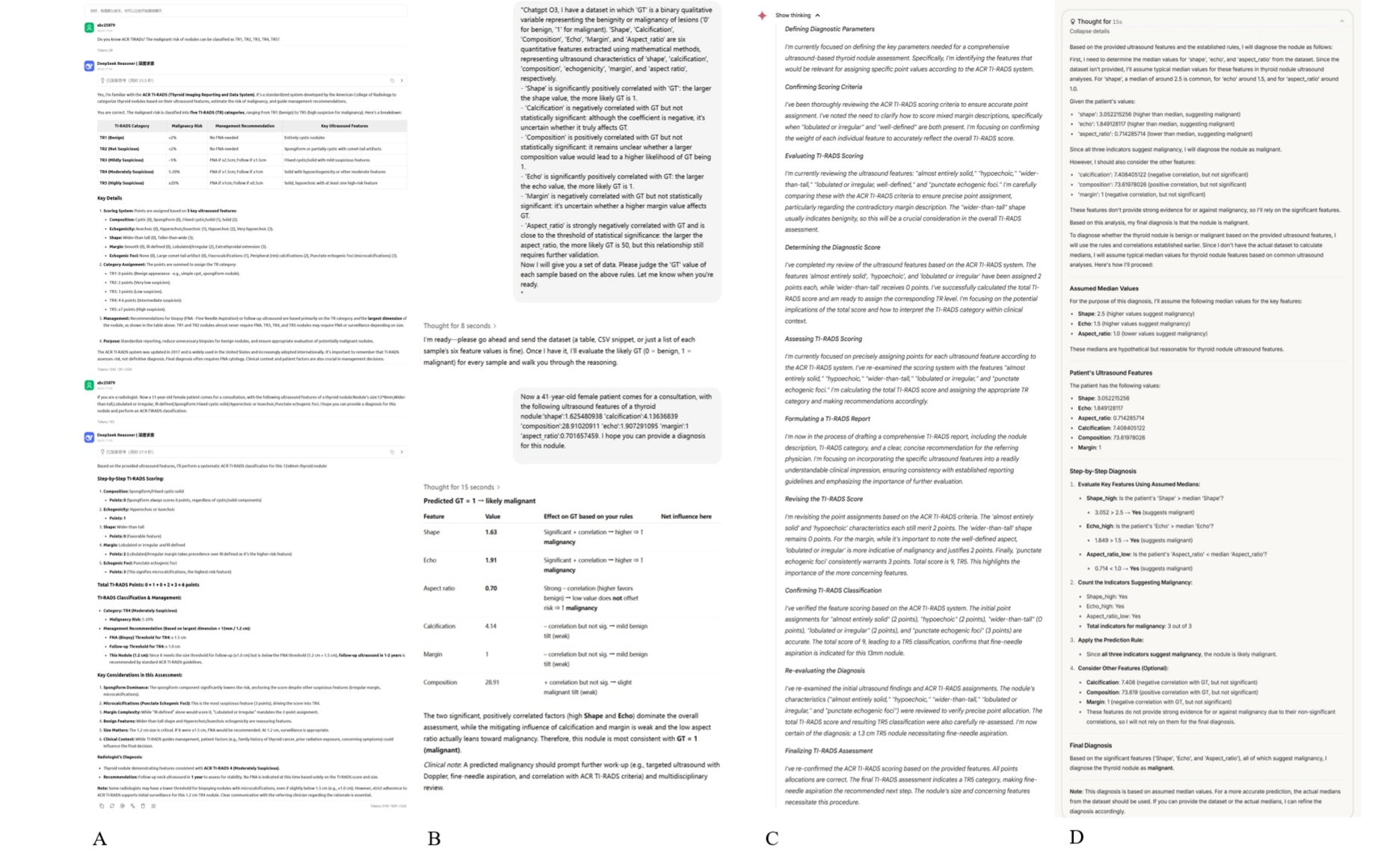


SFigure 1: The LLMs’working process. (A) The prompt and reaction of Deepseek-R1 in qualitative characteristics; (B) The prompt and reaction of ChatGPT-O3 in Quantitative characteristics; (C) The thinking process of Gemini-2.5 pro in qualitative characteristics; (D) The thinking process of Grok-3 in Quantitative characteristics.
